# The n-10 Fatty Acids Family in the Lipidome of Human Prostatic Adenocarcinoma Cell Membranes and Extracellular Vesicles

**DOI:** 10.3390/cancers12040900

**Published:** 2020-04-07

**Authors:** Carla Ferreri, Anna Sansone, Sandra Buratta, Lorena Urbanelli, Eva Costanzi, Carla Emiliani, Chryssostomos Chatgilialoglu

**Affiliations:** 1 Istituto per la Sintesi Organica e la Fotoreattività, Consiglio Nazionale delle Ricerche, Via P. Gobetti 101, 40129 Bologna, Italy; anna.sansone@isof.cnr.it (A.S.); chrys@isof.cnr.it (C.C.); 2 Department of Chemistry, Biology and Biotechnology, University of Perugia, Via del Giochetto, 06122 Perugia, Italy; sandra.buratta@unipg.it (S.B.); lorena.urbanelli@unipg.it (L.U.); eva.costanzi@studenti.unipg.it (E.C.); carla.emiliani@unipg.it (C.E.)

**Keywords:** sebaleic acid, sapienic acid, positional fatty acid isomer, trans geometrical isomer, extracellular vesicle lipidome, desaturase enzyme, elongase enzyme, lipidomics

## Abstract

A new pathway leading to the n-10 fatty acid series has been recently evidenced, starting from sapienic acid, a monounsaturated fatty acid (MUFA) resulting from the transformation of palmitic acid by delta-6 desaturase. Sapienic acid has attracted attention as a novel marker of cancer cell plasticity. Here, we analyzed fatty acids, including the n-10 fatty acid contents, and for the first time, compared cell membranes and the corresponding extracellular vesicles (EV) of two human prostatic adenocarcinoma cell lines of different aggressiveness (PC3 and LNCaP). The n-10 components were 9–13% of the total fatty acids in both cancer cell lines and EVs, with total MUFA levels significantly higher in EVs of the most aggressive cell type (PC3). High sapienic/palmitoleic ratios indicated the preference for delta-6 versus delta-9 desaturase enzymatic activity in these cell lines. The expressions analysis of enzymes involved in desaturation and elongation by qRT-PCR showed a higher desaturase activity in PC3 and a higher elongase activity toward polyunsaturated fatty acids than toward saturated fatty acids, compared to LNCaP cells. Our results improve the present knowledge in cancer fatty acid metabolism and lipid phenotypes, highlighting EV lipidomics to monitor positional fatty acid isomer profiles and MUFA levels in cancer.

## 1. Introduction

**Figure 1:**
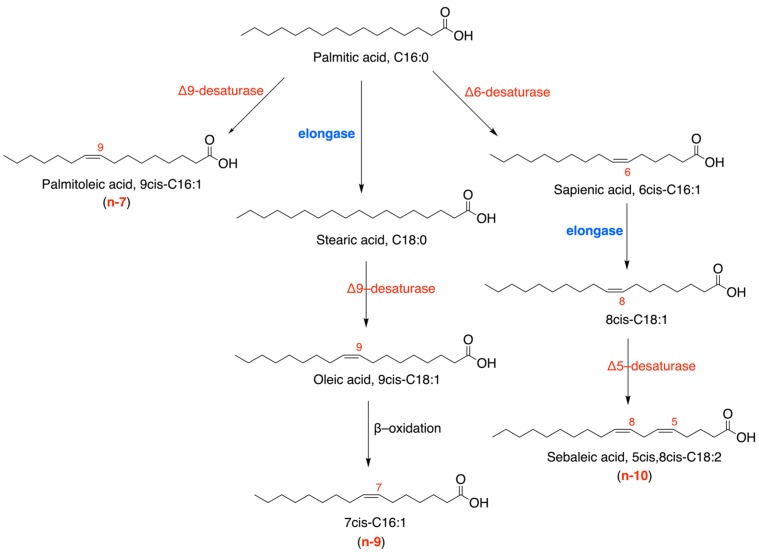
Main structures of the C16 monounsaturated fatty acids (MUFA) family and their biosynthetic pathways, starting from palmitic acid.

Lipid biosynthesis and cancer cell growth are strongly connected to each other in multiple aspects of replication, signaling and energy metabolism. Phospholipids are primary elements for cell membrane formation to ensure rapid duplication involved in carcinogenesis and invasiveness [1,2]. Follow-up of lipid metabolism in cancer cells clarified that not only a big quantity of lipids but also a specific quality of fatty acids is needed to provide structural and functional roles to cell membranes. In particular, after biosynthesis of two saturated fatty acids (SFA), palmitic (C16:0) and stearic acids (C18:0), monounsaturated fatty acids (MUFA) must be formed by delta-9 desaturase with the formation of palmitoleic (9cis-C16:1) and oleic (9cis-C18:1) acids, to gain cell membrane fluidity [3,4]. Such membrane status triggers a cascade of proliferation signals maintaining stemness, tumor formation and metastasis in breast, colon and prostate cancers [5,6]. Recent attention has been given to the hexadecenoic MUFA family (C16:1) composed by the positional and geometrical isomers depicted in Figure 1. As shown in Figure 1, C16-derived MUFAs belong to three different fatty acid series, namely the n-7, n-9 and n-10 series, all formed from palmitic acid, thus creating structural, biochemical and biological diversities. They attracted interest both for their unambiguous identification methodology, to be used in biological samples, and for their intrinsic biological activities. The first and most studied is the n-7 component, palmitoleic acid, evidenced by Cao and Hotamisligil for its lipokine-like activities [7]. Later on, its mitogen activity was reported [8] as well as its metabolism into an active compound in phagocytic cells [9]. This fatty acid is a known biomarker of desaturase activity in obesity [10] and risk of coronary heart disease found in the CAREMA (Cardiovascular Registry Maastricht) cohort study [11]. In plasma lipoprotein fractions (triglycerides, cholesteryl esters and phospholipids) of healthy subjects, we identified and quantified palmitoleic acid and, for the first time, its positional isomer of the n-10 series, sapienic acid (6cis-16:1), together with their corresponding trans geometrical isomers (9trans-C16:1 and 6trans-C16:1) [12]. We also described that morbidly obese subjects have statistically significant lower sapienic acid levels in plasma cholesteryl esters than healthy controls [13]. On the other hand, we observed that trans geometrical isomers of C16 MUFAs can be used as valuable biomarkers of the endogenous free radical-based isomerization occurring during oxidative stress metabolism [14], since they are not relevantly present in foods.

It is worth noting that a high sapienic/palmitoleic acid ratio was observed only in primary sebocytes undergoing differentiation and lipogenesis (pediatric source), thus becoming similar to adult sebocytes [15]. Moreover, sapienic acid formation increases for the delta-9 desaturase inhibition [5] or its reduced transcription, as well as for a decreased presence of linoleic acid, such as in the case of an accelerated beta-oxidation in sebocytes, as thoroughly discussed by Prouty and Pappas [16]. For the first time, we reported sapienic acid in the cell membrane phospholipids of a human cancer cell line (Caco-2) and, upon sapienic supplementation, we identified its metabolism, to 8cis-C18:1 and 5cis, 8cis-C18:2 (sebaleic acid) (see Figure 1) [17]. By using Laurdan two-photon microscopy, we demonstrated the change of fluidity features, comparing supplementations of the two positional isomers, palmitoleic and sapienic acids, to Caco-2 cells [17]. Sebaleic acid is a positional isomer of the n-6 polyunsaturated fatty acid (PUFA) linoleic acid (9cis,12cis-C18:2) and deserves a careful consideration, since it is the only endogenously formed PUFA, whereas the n-6 and n-3 C18 PUFA are essential fatty acids (EFA) for humans. Moreover, sapienic acid was recently indicated as a marker of cancer plasticity because of its high levels found in several cancer cell lines, mouse hepatocellular carcinoma, and primary human liver and lung carcinomas [18]. In this context, we envisaged that in the lipidome of cell-derived extracellular vesicles (EVs), the n-10 fatty acids are not described. 

EVs are membrane-enclosed structures that are released by every type of cells, including cancer cells [19]. They are classified into two main classes, i.e., exosomes, originating from the endosomal system and microvesicles, originating from the plasma membrane, although current separation protocols do not allow for separating exosomes from microvesicles, but only for separating small from large vesicles [20]. EVs carry all classes of macromolecules (lipids, proteins and nucleic acids, namely miRNA) and can be retrieved in every fluid of the body, and in the context of cancer biology, they carry oncoproteins and oncogene nucleic acid fragments. In addition, evidence has been provided that EVs have crucial roles in cancer development, including pre-metastatic niche formation and metastasis, and can be used as diagnostic and prognostic markers [21]. EV lipidome analyses are reported using shotgun mass spectrometry, i.e., under conditions that are not able to distinguish fatty acids of the same molecular mass and different position/geometry along the hydrocarbon chain [22,23]. 

Based on these premises, we decided to perform untargeted lipidomic analysis, with specific attention to the n-10 fatty acid series, in two human prostate cancer cell lines with a high and low metastatic potentials, PC3 and LNCaP (PC3: prostate cancer; LNCaP: Prostate Derived From Metastatic Site: Left Supraclavicular Lymph Node) respectively, widely studied for whole-genome sequencing [24], and their released EVs [25]. In the present work, we use analytical detection by GC (gas chromatography) and GC/MS (gas chromatography/mass spectrometry) under known conditions that allow for separation and characterization of positional/geometrical isomers [17]. In parallel, elongation and desaturation enzyme expressions were determined in the two cell lines. 

We anticipate that consistent differences will be found in the two cell lines and their EVs regarding the n-10 fatty acid series, foreseeing further development for markers associated to cancer metabolism and metastatic potential. These findings expand the knowledge of fatty acid isomers in cancer lipid phenotypes that are important to be integrated in cancer–omic research.

## 2. Results

### 2.1. Fatty Acid Profiles of PC3 and LNCaP Cell Lines 

Cultivation of PC3 and LNCaP cell lines was performed following literature procedures [26] and EVs were isolated from cell culture medium, as described below in the Materials and Methods Section. Cell membrane pellets of PC3 (*n* = 8) and LNCaP (*n* = 8), and corresponding PC3-EVs (*n* = 8) and LNCaP-EVs (*n* = 8), were first examined for the lipid classes, identifying phosphatidylethanolamine (PE), phosphatidylserine (PS) and phosphatidylcholine (PC), together with cholesterol (CHO) and sphingomyelins (SM), by appropriate methods and standard references (Table S1 in the Supplementary Information). Phospholipids (PE, PS, PC) have fatty acid residues, and under known conditions of transesterification, give fatty acid methyl esters (FAME) [12,13,17]. Fatty acids were recognized by appropriate standards and quantified by GC analysis as µg/mL (Table S2 of the Supplementary Information; see Figures S1–S3 in the Supplementary Information as representative examples of GC chromatograms and peak identification). From these quantitative data, the percentages of each fatty acid in the total fatty acid content of PC3 and LNCaP cell membrane phospholipids and their EVs were obtained (% relative quantities, % rel. quant.) and the values are shown in Table 1, and in Table 2 as mean ± standard error of the mean (S.E.M) of *n* = 8 replicates. First of all, we consider the fatty acid composition of cell membrane phospholipids shown in Table 1 with a particular interest for n-10 fatty acids, following sapienic acid to sebaleic acid transformation, in order to envisage differences of fatty acid composition between the two cell types. Interestingly, the n-10 fatty acids are present in both cell types, reaching >12% of the total fatty acids. Both cell types showed that the membrane content of sapienic acid is >3 times higher than palmitoleic acid, with palmitoleic acid being significantly lower in LNCaP than in PC3. As far as the trans geometrical isomers are concerned, the trans isomer of sapienic acid was detected in a significantly higher quantity in LNCaP cells than in PC3. Regarding other fatty acids of the membrane profile, linoleic acid (C18:2 n-6) was found lower and arachidonic acids (C20:4 n-6) were found higher in LNCaP than in PC3. 

### 2.2. Characterization, Lipid Distribution and Fatty Acid Profiles of EVs Released from PC3 and LNCaP Cells 

EVs were isolated from the cell culture media of the two prostate cancer cell lines, PC3 and LNCaP, using a differential centrifugation method [27,28], and further characterized by electron microscopy and Western blot analyses (Figure S4 in the Supplementary Information). The amount of released EVs in each preparation was assessed measuring the total protein content and finding similarity for the two cell types (~3 µg/1 × 10^6^ cells) (Figure S4A in the Supplementary Information). The morphology of purified EVs was examined using scanning electron microscopy (SEM) (Figure S4B in the Supplementary Information). The images revealed the presence of round cup-shaped vesicles with diameters ranging from 50 to 100 nm, thus giving direct evidence of the EV size and morphology as required for their characterization. In addition, the expression of EV marker proteins was evaluated by Western blot (Figure S4C in the Supplementary Information). Isolated EVs and parental cells were probed with positive markers such as tetraspanins (CD9 and CD81) and Alix (protein involved in EV biogenesis) in agreement with guidelines [20]. To exclude contamination with intracellular structures, Western blots for calnexin (marker of endoplasmic reticulum) and actin were also performed. Results showed that EVs from both cell lines contained the two tetraspanins and Alix, whereas calnexin and actin were not present (Supplementary Figure S4). These results indicated that our EV preparations were devoid of contamination with endoplasmic reticulum or cytoskeletal components and were enriched of vesicles having a size typical of small EVs, consistent with exosomes and/or small membrane microvesicles [20,28]. Moreover, the determination of lipid classes in cells and in their corresponding EVs was performed and the comparison of the lipid distribution showed that EVs have a peculiar composition as compared to their parental cells (Table S1 in the Supplementary Information). In both cell lines, the main difference is a higher percentage of cholesterol (CHO, ≈13%) and phosphatidylserine (PS, ≈26%) in EVs with respect to cells (≈5%), accompanied by a lower percentage of phosphatidyl ethanolamine (PE) and phosphatidyl choline (PC) (Supplementary Table S1). This finding suggests that EVs may generally express a different lipid composition with respect to their originating cells. These results are in agreement with previous studies on lipid composition of EVs, demonstrating that vesicles from different cell types are enriched in CHO, PS and glycosphingolipids with respect to parental cells [22,29,30].

The EVs fatty acid composition of PS, PE and PC was determined as described in the Materials and Methods Section, and Table 2 shows the % relative quantities (% rel. quant.) of the fatty acids. The corresponding quantities in µg/mL are detailed in Table S2 of the Supplementary Information. The n-10 family was present in LNCaP-derived EVs with percentages similar to the corresponding cell membranes (>12%), and again, in both EVs, sapienic acid was higher than palmitoleic acid. Interestingly, in PC3-derived EVs, the contents of 8*cis*-C18:1 and sebaleic acid were significantly lower than in EVs from LNCaP, thus, as a result, the total n-10 fatty acid content significantly decreased. It was also found that EVs present a significantly different fatty acid distribution among SFA, MUFA and PUFA families comparing the two cell types, showing that in PC3-derived EVs, the MUFA content is increased (+10%), in particular with the increase of oleic acid (9*cis*-C18:1), and SFA and PUFA families decreased (~4%–5% each) compared to LNCaP. As far as PUFAs are concerned, in PC3-EVs, the n-6 family presents significantly less arachidonic acid and the n-3 family presents all of its components significantly decreased, thus bringing the n-6/n-3 ratio close to 2. The trans isomer of sapienic acid was also significantly higher in LNCaP-EVs, as it was shown for the cell membrane phospholipids. Overall, the differences between the two cell lines were more evident in EVs than in cell membranes.

It is worth recalling that with our analytical methodology, all samples underwent the treatment of dimethyl disulfide (DMDS) derivatization, as previously reported [12,13,17], to unambiguously individuate the position of the double bonds along the chain, thus assessing the presence of the C16 MUFA isomers and the n-10 C18 MUFA and PUFA. In the Supplementary Information, Figure S3 shows representative GC chromatograms related to this identification.

### 2.3. Characterization of Fatty Acids-Related Enzyme Expressions in PC3 and LNCaP Cells

**Figure 2:**
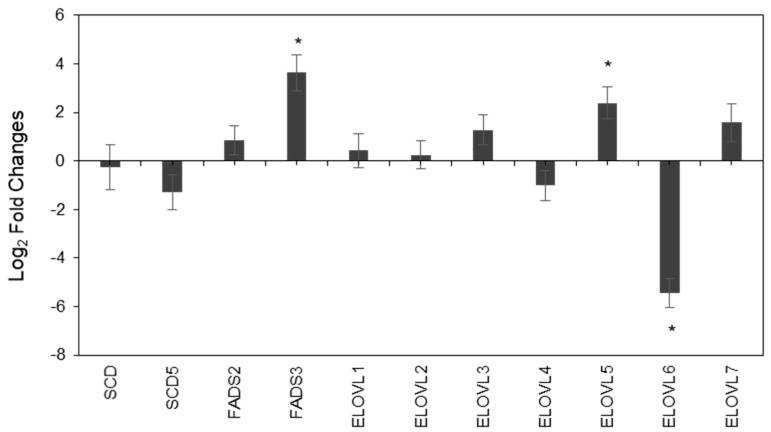
Evaluation of desaturase and elongase expression in PC3 cells by qRT-PCR (quantitative real Time polymerase chain reaction). X-axis presents 4 desaturase and 7 elongase enzymes. Y-axis represents the log2 (fold-change) of PC3 with respect to LNCaP cells. Fold-change was calculated by the comparative CT method (ΔΔCT) using glyceraldehyde 3-phosphate dehydrogenase (GAPDH) as control for ΔC_t_ and calculating ΔΔCt by subtracting ΔC_t_ for PC3 to ΔC_t_ for LNCaP.

We also evaluated the expression of enzymes involved in fatty acids biosynthesis in PC3 and LNCaP cells by qRT-PCR, with attention to desaturases and elongases. In the Supplementary Information, the list of primers used for this analysis is reported in Table S3 (see Supplementary Information). The elongation enzymes, involved in the addition of two carbon atom units to palmitic acid, are characterized by the acronym ELOVL (elongation of very long-chain fatty acids), whereas the desaturation enzymes, involved in the double bond formation, are indicated either by the acronym SCD (stearoyl CoA desaturase) or FADS (fatty acid desaturase). In Figure 2, the fold-increase or decrease of enzyme expression in PC3 cells is reported with respect to LNCaP cells. We found that FADS3 is expressed at a higher level in PC3 cells. Besides, FADS1 (not indicated in Figure 2) is also expressed at a higher level in PC3. It was possible to amplify FADS3 from PC3 cells in standard conditions, whereas only a higher amount of cDNA (×10) and less stringent amplification conditions allowed for amplification from LNCaP (data not shown). FADS3 is clustered with family members FADS1 and FADS2 at 11q12-q13.1260 [31]. It is the less characterized member of the desaturase family and even if it is likely to be a desaturase, its exact function remains elusive. It has been implicated in delta-13 desaturation of trans-vaccenic acid (11*trans*-C18:1) [32]. However, FADS3 knockout mouse model confirmed lower desaturase activity, whereas no delta-13 desaturation of vaccenic acid was observed [33]. More recently, evidence has been provided that FADS3 is involved in delta-14 sphingoid base desaturation [34], but this finding needs additional confirmatory studies. FADS1 has a delta-5 desaturase activity, although recent evidence of a delta-7 desaturase activity has also been reported [35]. It is involved in the synthesis of critically important PUFA [36]. FADS3 is an extensively spliced protein, with at least 8 alternative transcripts that are conserved [36]. Higher levels of FADS1 and FADS3 in PC3 would be expected to be associated with a higher level of desaturation. Looking at the results of the cell membranes, no significant differences of total MUFA and PUFA levels, except for the higher level of palmitoleic acid (9*cis*-C16:1) and lower level of n-6 linoleic acid, were detected in PC3 compared to in LNCaP. Instead, for their released EVs, a significantly higher level of total MUFA, in particular, oleic acid, was observed in PC3. 

The unclear function of the enzyme isoforms (and genetic polymorphisms) makes it difficult to speculate about the consequences of its higher expression in PC3 cells. Besides, recent findings also suggest that FADS3 might be related to sphingolipid metabolism. As for FADS1, a high number of the functional variants have also been reported to be associated with this gene and their role in affecting long-chain PUFA biosynthesis is currently unclear [37]. Besides, FADS1 and FADS2 compete for the same substrates and both cell types show similar levels of FADS2.

Analysis of elongase transcripts showed that ELOVL5 was expressed at a higher level in PC3 than LNCaP, whereas the level of ELOVL6 was remarkably lower. ELOVL5, together with ELOVL2, is involved in PUFA elongation. ELOVL5 is specific for 18 and 20 carbon fatty acids, whereas ELOVL2 is a C20-24 PUFA elongase [38]. On the other hand, ELOVL6 prefers SFA and MUFA as substrates, elongating fatty acids with 12, 14 and 16 carbons. Similar preferences are shown by ELOVL1, ELOVL3 and ELOVL7, whereas ELOVL4 prefers SFA and very long-chain PUFA, with 28 to 38 carbons. Consequently, despite its remarkably lower level in PC3 cells, ELOVL6 function in fatty acid elongation may be compensated by ELOVL1, ELOVL3 and ELOVL7, which possess overlapping activity and are comparably expressed between the two cell types [36].

It must be also considered that fatty acids from membrane phospholipids may not only reflect the expression level of enzymes involved in their biosynthesis, but also the activity of enzymes involved in fatty acid remodeling of membrane phospholipids, such as lysophospholipid acyltransferases [39].

## 3. Discussion

The formation of the n-10 fatty acid series is an emerging pathway in cancer cell metabolism, providing MUFA and the de novo PUFA component, sebaleic acid (see Figure 1). Sapienic acid, which is the precursor of the n-10 series, was detected for the first time in sebocytes [15], and later, it was found in human blood circulating lipids and red blood cell membranes [12,13] as well as in a Caco-2 cell line [17]. It is interesting to note that sapienic acid in Caco-2 cell membrane phospholipids corresponded to 1.6% of the total recognized FA, and the sapienic/palmitoleic ratio was in favor of the latter (1.6/7 = 0.23). By monitoring the Caco-2 cell membrane lipidome after sapienic acid supplementation, it was shown that the n-10 fatty acid metabolism occurs immediately and its metabolites are incorporated as membrane phospholipid fatty acids, i.e., in 30 min as 8cis-C18:1 and in 1 hour as the PUFA sebaleic acid. In the present work, for the first time, we examined PC3 and LNCaP cell lines for the n-10 MUFA and PUFA, finding them at a very high percentage (>12%). This is an important result highlighting such metabolism in these cell lines compared to the colon cancer cell line. Indeed, PC3 and LNCaP cell lines present high levels of sapienic acid (>7%) and a sapienic/palmitoleic acid ratio in favor of the former (ca. 3.5). It is worth noting that in our previous Caco-2 cell line study [17], we found an initial lower n-10 asset, but the sapienic acid supplementation reverted it to reach a sapienic/palmitoleic acid ratio > 2.3. It is worth underlining that the palmitic acid partitioning between delta-6 and delta-9 desaturase enzymes is also under investigation for the genetic polymorphisms [40,41]. In a recent paper by Vriens et al. [18], sapienic acid was reported in several human cancer cell lines, with increased levels compared to healthy cells. Sapienic acid was discussed as a sign of increased plasticity of cancer cells, however these authors did not follow-up on the metabolism of sapienic acid up to sebaleic acid. We can argue that as a sign of plasticity, the whole n-10 fatty acid series must be followed up, and more importantly if we consider the formation of sebaleic acid as an endogenous PUFA that gives a decisive contribution to fluidity and adaptability features of eukaryotic cells. While the endogenous production of PUFAs cannot occur in eukaryotic cells without dietary supplementation of precursors, sebaleic acid is the only PUFA formed de novo in human cells. Its fate must be addressed in further work, also in order to be associated to a specific cancer cell phenotype, as well as to signaling pathways or plasticity features. It is important to recall that de novo lipogenesis and membrane saturation are interpreted as a “resistance” outcome in cancer cell metabolism [42]. The significant increase of the geometrical trans isomer of sapienic acid in LNCaP cell membranes and EVs can be accounted for by an endogenous free radical-based cis-trans isomerization occurring under cellular stress [13,14]. The significance of such data in cancer must be deepened in further work. 

Since the LNCaP cell line is known to have a less invasive profile than PC3, it is interesting to highlight the observed differences of their membrane lipidomes and EVs: (a) PC3 have a significantly high n-9 MUFA palmitoleic acid in cell membrane and an increase of n-9 oleic acid and total MUFA family in EVs, both considered as invasiveness markers associated with enhanced desaturase enzymatic activities [3,4]. (b) In PC3-EVs, the total SFA and PUFA contents are significantly lower than LNCaP-EVs, and among PUFA levels in PC3 the n-3 content was particularly reduced. The latter could indicate loss of molecular factors and related signaling that are known to control prostate cancer invasiveness and aggression [43,44,45]. It is worth recalling at this point that the mass spectrometry tools for lipidomic research and EVs analysis have already evidenced important differences that can be used for diagnostic purposes [22,23]. In our work, we could properly address the detection of the n-10 fatty acids that are positional isomers of MUFA and PUFA present in naturally occurring lipids. 

The n-10 fatty acid family contributes to the increase of MUFA biosynthesis and the enrichment found in EVs could play a role when the transfer of biologically active lipids and lipid metabolites is used as a mechanism to affect the cancer microenvironment. It will be extremely important to target further research in EVs of other cell lines toward the correlation between de novo synthesized n-10 fatty acids and the presence of angiogenesis, growth and other metabolism-affecting factors. Further, the EV cargos enriched by the n-10 family could also contribute to the transport of MUFA and PUFA to hypoxic tumoral tissues, where the desaturation is possibly decreased, allowing the subsequent utilization of these fatty acids as substrates for fatty acid oxidation [46]. Another point of interest concerns the relationship between membrane fluidity and metastatic potential, which is not the target of this work. Although the presence of the n-10 fatty acid family in membrane phospholipids has been proven by us [17] and others [18], and some information was given for membrane fluidity changes [17], the specific contributions to the lipid asset must be determined, also in view of therapeutic strategies and anticancer drugs able to modulate membranes, that are under development [47]. It is worth noting that recently, sapienic and 8*cis*-C18:1 were found in several cancer cell lines, however the PUFA sebaleic acid was not followed up [18]. Since lipids give an important contribution to cancer metabolic plasticity, our findings on n-10 metabolism to PUFA and their presence in EVs of prostate cancer cells propose novel aspects for further investigations in other cell lines. 

Using PCR analysis for the enzyme expressions in the two different cell lines, we individuated a higher desaturase expression in PC3 cells than in LNCaP, that could combine with the higher level of palmitoleic acid (9*cis*-C16:1), as well as with the higher levels of linoleic acid and lower levels of arachidonic acid found in PC3 cell membranes. Since palmitoleic acid is a marker of endogenous formation of the double bond in position 9 and linoleic acid is transformed by delta-6 desaturase, these fatty acid levels could have an indirect connection with the enzymatic expressions that were found. As a matter of fact, the significant low levels of arachidonic acid in the most aggressive PC3 cell line could not mean a diminished biosynthesis, but could be a cellular response producing the release of arachidonic acid with its involvement in oxidative and signaling processes, as previously described by some of us for pancreatic cell lines [48]. On the other hand, the released EVs of the two cell lines showed a significantly higher level of MUFA in PC3 than in LNCaP, which reveals the possibility that the cellular desaturase increase is then reflected into the corresponding EV composition, also to fulfil the role of cargos, as explained above. This can also be true for the reduced elongation activity (ELOVL6) that affects SFA levels in PC3-EVs. The increase of MUFA and decrease of SFA were evaluated in fibroblasts exposed to H-Ras and this was associated to senescence [49], although in that case, the identification of the MUFA families was restricted to oleic and palmitoleic acids. For FADS3, recent research highlighted its involvement for the biosynthesis of long-chain PUFA, where it was shown that FADS3 desaturase activity reduces elongase expression and activity [36]. Our results suggest that silencing experiments are needed in order to gain a deep understanding of the lipid enzymes that influence the n-10 fatty acid series, as well as the palmitic acid partition between delta-9 and delta-6 desaturases with corresponding sapienic/palmitoleic ratios, taking into account that such enzymes are targets of anticancer strategies [5].

Further efforts are needed to gather a full understanding of the fatty acid balance reached in specific health conditions, and we believe that the lipid asset including n-10 series represents a relevant step forward, in particular to fight cancer invasiveness and survival. 

## 4. Materials and Methods

Sapienic acid methyl ester, 8*cis*-18:1 methyl ester and sebaleic acid methyl ester were purchased from Lipidox (Lidingö, Sweden). cis and trans fatty acid methyl esters (FAME), dimethyl disulphide, iodine, cholesterol, sphingomyelin and formic acid acid were purchased from Sigma-Aldrich (San Louis, MO, USA), and used without further purification. Chloroform, methanol, isopropanol, diethyl ether and n-hexane were purchased from Baker (HPLC grade) and used without further purification. POPC (1-palmitoyl-2-oleoyl-sn-glycero-3-phosphocholine); POPE ( 1-palmitoyl-2-oleoyl-sn-glycero-3-phosphoethanolamine); POPS ( 1-palmitoyl-2-oleoyl-sn-glycero-3-phosphoserine) were purchased from Larodan and used without further purification.

Silica gel analytical thin-layer chromatography (TLC) was performed on Merck silica gel 60 plates, 0.25 mm thickness, and spots were detected by spraying the plate with cerium ammonium sulfate/ammonium molybdate reagent. 

Fatty acid methyl esters (FAME) were analyzed by GC (Agilent 6850, Milan) in splitless mode, equipped with a 60 m × 0.25 mm × 0.25 µm (50%-cyanopropyl)-methylpolysiloxane column (DB23, Agilent, USA), and a flame ionization detector, with the following oven program: temperature started from 165 °C, held for 3 min, followed by an increase of 1 °C/min up to 195 °C, held for 40 min, followed by a second increase of 10 °C/min up to 240 °C, and held for 10 min. A constant pressure mode (29 psi) was chosen with helium as the carrier gas. Methyl esters were identified by comparison with the retention times of authentic samples. LOD (limit of detection) and LOQ (limit of quantitation) values of the GC instrument were described in previous works [17]. The FAME are expressed as µg/mL (mean ± S.E.M.) using the calibration procedures previously described [17], and are reported in Table S2 in the Supplementary Information as well as in quantitative relative percentages (mean ± S.E.M) (Table 1 and Table 2 in the main text).

Dimethyl disulphide adducts of FAME were analyzed by GC-MS (Thermo Scientific Trace 1300) equipped with a 15 m × 0.25 mm × 0.25 µm 5% phenyl methyl polysiloxane column (Thermo Scientific™ TraceGOLD™ -**SQC**) with helium as the carrier gas, coupled to a mass-selective detector (Thermo Scientific ISQ, Waltham, MA, USA) with the following oven program: temperature started at 80 °C, maintained for 2 min, increased at a rate of 15 °C/min up to 140 °C, increased at a rate of 5 °C/min up to 280 °C and held for 10 min.

Phospholipid classes were analyzed by HPLC (Agilent 1200, Santa Clara, CA, USA) equipped with RP18 (Macherey-Nagel EC 150/4.6 Nucleodur C18 HTEC,5µM) using the following isocratic condition: H_2_O + 0.2% HCOOH/MeOH/ 2-propanol 10/20/70, detector UV 203. The phospholipids were identified by comparison with the retention times of authentic samples. The values are expressed in µg/mL.

### 4.1. Cell Culture

The human prostatic carcinoma cell lines, PC-3 and LNCaP, were obtained from America Type Culture Collection 8ATCC, Manassas, VA, USA). Cells were grown in RPMI 1640 medium supplemented with 10% (v/v) heat-inactivated fetal bovine serum (FBS), 2 mM glutamine, 100 µg/mL streptomycin, in a humidified atmosphere containing 5% CO_2_ at 37 °C. For experimental purposes, cells (5 × 10^6^ cells/10 mL medium) were seeded in 75 cm^2^ flasks and, after reaching sub-confluence, the culture medium was carefully removed and the cells were washed twice with phosphate-buffered saline (PBS). Then, cells were maintained for 24 h in serum-free medium. At the end of this period, cell culture media were collected to isolate EVs. Cells were recovered and counted by Countess (Countess™ Automated Cell Counter, C10227, Invitrogen, Carlsbad, CA, USA). Cell viability was assessed by Trypan Blue stain exclusion. 

### 4.2. EV Isolation

EVs were isolated from cell culture media (30 mL) by a differential centrifugation protocol [26,27]. Briefly, media were subjected to low-speed centrifugations to remove cells, cell debris and large EVs (300× *g* for 10 min; 2000× *g* for 20 min; 10,000× *g* for 30 min; Rotor F34-6-38 and Centrifuge 5804R). The supernatants were ultracentrifuged at 100,000× *g* (Rotor 60 TI Fixed Angle Rotor and Beckman L8-70 MR Ultracentrifuge) for 70 min to pellet EVs, which were washed in PBS and centrifuged again at 100,000× *g* for 70 min (MLS-50 rotor and an Optima Max ultracentrifuge). EV pellets were re-suspended in small volume of PBS (150 µL) and used for further analyses. Protein concentration was determined by Biorad assay, using bovine serum albumin as standard.

### 4.3. Scanning Electron Microscopy

For scanning electron microscopy (SEM) examination, EVs were fixed in 2.5% glutaraldehyde for 15 min at room temperature, washed twice with a large volume of water using Vivaspin concentration devices (300,000 Da cut-off), then sedimented on glass coverslips and allowed to dry at room temperature. SEM images were obtained using a field emission gun electron scanning microscope (LEO 1525 Zeiss; Thornwood, NY, USA) after Cr metallization using a high-resolution sputter Q150T ES-Quorum apparatus (24 s sputter at a current of 240 mA). Chromium thickness was ~10 nm.

### 4.4. Western Blotting 

For Western blotting analyses, cells were recovered, washed twice with PBS, and centrifuged again. Approximately 3 × 10^6^ cells were lysed at 4 °C in RIPA (Radio-Immune Precipitation Assay) buffer (50 mM Tris-HCl pH 8, 150 mM NaCl, 1% (v/v) NP-40, 0.1% (w/v) SDS (sodium dodecylsulfate), 0.5% (w/v) sodium deoxycholate) in the presence of a protease inhibitor mixture. Insoluble material was removed by centrifugation at 13,000× *g* for 10 min at 4 °C.

Aliquots of cell lysates (10–30 μg) or EVs (5 μg) were mixed with sample buffer 5× (1 M Tris-HCl pH 6.8, 5% (w/v) SDS, 6% (v/v) glycerol, 0.01% (w/v) Bromophenol blue) without DTT (dithiothreitol) (non-reducing conditions, used for CD9 detection according to the manufacturer’s instructions) or with 125 mM DTT (used for the other antibodies). Samples were boiled for 5 min, electrophoresed on 12% acrylamide gel and electrotransferred to PVDF (polyvinylidene fluoride) membrane. After 30 min incubation at room temperature with blocking buffer (5% BSA in TBS-Tween), membranes were incubated overnight with the following primary antibodies: goat polyclonal anti-Alix (Santa Cruz, USA), mouse monoclonal anti-CD9 (Abcam, Cambridge, UK), mouse monoclonal anti-CD81 (Santa Cruz, USA), goat polyclonal anti-calnexin (Sigma-Aldrich, St Louis, MO, USA) and anti β-actin (Sigma-Aldrich).

### 4.5. Quantitative PCR

RNA was extracted using Trifast reagent (Euroclone), according to the manufacturer’s instructions. 1 μg of RNA was reverse-transcribed into cDNA using random hexamers and SuperScript II Reverse Transcriptase (Life Technologies, Carlsbad, CA, USA). cDNA was used to determine transcript levels by qRT-PCR in a StepOne RT-PCR machine (Applied Biosystems, Foster city, CA, USA) using SYBR® Select Master Mix (Life Technologies). Primers used are listed in Supplementary Table S3. Glyceraldehyde 3-phosphate dehydrogenase (GAPDH) used as endogenous control was amplified with primers 5’-GAG AAG GCT GGG GCT CAT TT (forward) and 5’-AGT GAT GGC ATG GAC TGT GG (reverse). Data were analyzed using the ΔΔCT method. ΔC_t_ was calculated subtracting the average C_t_ value of GAPDH as control to the average C_t_ value of each transcript for PC3 and LnCaP. ΔΔC_t_ is the difference between the ΔC_t_ for each transcript for PC3 and the ΔC_t_ of each transcript for LnCaP as control. The reported fold expression, expressed as RQ (relative quantity), was calculated by 2^−ΔΔCt^. The analysis was repeated three times in triplicate. The mean ± standard deviation (SD) of a representative experiment is reported (* *p* < 0.05). 

### 4.6. Membrane Lipid Characterization, Extraction and Fatty Acid Analysis of PC3 cells, LNCaP Cells and Their Corresponding Extracellular Vesicles.

To the pellets of PC3 cells (3 × 10^6^ cells) and LNCaP cells (3 × 10^6^ cells), tridistilled water (1 mL) was added and samples were centrifuged at 10,000× *g* for 15 min at 4 °C. The supernatant was discarded and the pellets were re-suspended in 1 mL of tridistilled water. An aliquot of 20 µL was used for the HPLC analysis of phospholipid classes (Table S1 in the Supplementary Information), and the rest of the suspension was extracted with 2:1 chloroform/methanol (4 × 4 mL) according to the Folch method [50]. The organic layers were dried on anhydrous Na_2_SO_4_ and evaporated to dryness. The total lipid extracts (0.6–0.7 mg) were analysed by thin layer chromatography (TLC) (eluent: *n*-hexane:diethyl ether 9:1) for their composition, confirming the presence of phospholipids and cholesterol as the main components. The extracts were converted to FAME (fatty acid methyl esters) by adding 0.5 M KOH in MeOH (0.5 mL). After 10 minutes, the reaction was quenched by brine (0.5 mL) and FAME were extracted with n-hexane (4 × 2 mL), dried on anhydrous Na_2_SO_4_ and evaporated to dryness. GC analysis was performed using standard references for peak identification and quantitation. Extracellular vesicles were worked up as described for cell samples, except for the centrifugation step, since the samples (300 µL/100 µg proteins) were added with tridistilled water (0.3 mL) and centrifuged at 100,000× *g* for 1.5 h at 4 °C in order to separate the pellet. 

### 4.7. Dimethyl disulfide (DMDS)Derivatization

The FAME mixture obtained from PC3 and LNCaP cells and EVs followed a previously described procedure for the derivatization and GC/MS analysis in order to proceed with the assignment of the double bond position [17]. Briefly, in a Wheaton vial containing FAME-dissolved *n*-hexane (50 μL), 70 μL of dimethyl disulfide and 2 drops of a 6% solution of iodine in diethyl ether were consecutively added. The reaction was stirred at room temperature for 1.5 h under an argon atmosphere. Then, 1 mL of *n*-hexane and 1 mL of a 5% aqueous solution of sodium thiosulphate were consecutively added. The organic phase was isolated, dried over anhydrous Na_2_SO_4_, and concentrated under a gentle stream of nitrogen, before the GC-MS analysis (Figure S3 in the Supplementary Information shows a representative example of GC analyses).

### 4.8. Statistical Analysis

For statistical analysis, the fatty acids were expressed in relative percentages and expressed as mean ± S.E.M (standard error of the mean). Statistical analysis was performed using GraphPad Prism 5.0 software (GraphPad Software, Inc., San Diego, CA, USA). We used the non-parametric unpaired *t*-test, two-tailed, with a 95% confidence interval. 

## 5. Conclusions

The detailed fatty acid characterization for each cancer cell type can provide interesting information, envisaging important applications not only for molecular biology studies but also for clinical and nutritional research applications. Fatty acids are a relevant part of the metabolism and the diet and can offer escaping strategies for cell survival. Since delta-6 desaturase partitioning between palmitic acid for sapienic acid formation and dietary PUFAs is not yet disclosed, the characterization of the fatty acid profiles and the contribution of n-10 series in specific cancer cell lines is relevant to fully understand metabolic pathways and individuate anticancer strategies. Indeed, this information could be useful to design lipid therapies with n-6/n-3 PUFA supplementation, tailored to balance the n-10 fatty acid formation. Fatty acid-based functional lipidomics with isomers detection can bring important information that is extendable to personalized molecular profiles and nutritional approaches for cancer patients. It is known that PUFA are regulators of several metabolic pathways and signaling related to cancer [51,52,53,54]; therefore, it is important to provide a complete scenario that helps in the overall comprehension of the balance between n-7, n-9 and n-10 fatty acid series with n-6 and n-3 dietary contribution. Our results are relevant to improve the present knowledge in cancer phenotype and diagnostics, highlighting EV lipidomics to monitor positional fatty acid isomer profiles and MUFA levels in cancer.

## Figures and Tables

**Table 1:** Fatty acid methyl esters (FAME) (% relative quantities, %rel. quant.) obtained from membrane phospholipids of two different prostate cancer cell lines.

FAME ^1^	PC3 (*n* = 8) ^2^	LNCaP (*n* = 8) ^2^
C14:0	3.26 ± 0.32	3.44 ± 0.17
C16:0	33.20 ± 1.33	33.60 ± 0.49
6*trans*-C16:1	0.36 ± 0.04	0.56 ± 0.03 **
6*cis-C*16:1 n-10	7.41 ± 0.35	7.81 ± 0.33
9 *cis*-C16:1 n-7	2.36 ± 0.17	1.96 ± 0.05 *
C18:0	10.92 ± 0.54	11.48 ± 0.25
9*trans*-C18:1	0.09 ± 0.01	0.11 ± 0.02
8*cis*-C18:1 n-10	5.98 ± 0.75	4.55 ± 0.12
9*cis*-C18:1 n-9	19.47 ± 0.72	18.62 ± 0.24
11*cis*-C18:1 n-7	3.92 ± 0.30	3.90 ± 0.08
5*cis*,8*cis*-C18:2 n-10	0.47 ± 0.03	0.52 ± 0.04
mono-*trans* C18:2 n-6	0.27 ± 0.05	0.24 ± 0.02
C18:2 n-6	2.48 ± 0.19	1.97 ± 0.08 *
C20:3 n-6	1.62 ± 0.16	1.73 ± 0.18
C20:4 n-6	2.86 ± 0.23	4.01 ± 0.14 ***
mono-*trans* C20:4	0.10 ± 0.01	0.11 ± 0.05
C20:5 n-3	0.47 ± 0.06	0.37 ± 0.07
C22:5 n-3	1.77 ± 0.19	1.53 ± 0.14
C22:6 n-3	2.98 ± 0.18	3.49 ± 0.20
SFA	47.38 ± 1.39	48.52 ± 0.46
MUFA	39.14 ± 1.13	36.84 ± 0.59
PUFA	12.65 ± 0.37	13.62 ± 0.42
n-6	6.96 ± 0.34	7.70 ± 0.31
n-3	5.22 ± 0.15	5.40 ± 0.38
n-6/n-3	1.34 ± 0.07	1.52 ± 0.19
n-10	13.86 ± 0.97	12.89 ± 0.42
Total *trans*	0.83 ± 0.07	1.02 ± 0.06

^1^ FAME = fatty acid methyl ester. Identified by standard references and quantified as described in the Materials and Methods Section. Values are obtained in µg/mL considering the gas chromatography (GC) peak areas recognized and calibrated with standard references (corresponding to >98% of the total peaks of the chromatogram). ^2^ PC3, prostate cancer and LNCaP, Prostate; Derived From Metastatic Site: Left Supraclavicular Lymph Node. Values are expressed in percentages relative to the sum of all the quantities of the recognized peaks ± standard error of the mean (S.E.M) from the analyses of *n* = 8 cell samples of each type; statistical significance is estimated: * *p*-value ≤ 0.045; ** *p-*value ≤ 0.001; *** *p*-value ≤ 0.0009. Details of the statistical analysis are reported in the Materials and Methods Section.

**Table 2:** Fatty acid methyl esters (FAME) (%rel. quant.) obtained from extracellular vesicles (EV) derived from two different prostate cancer cell lines.

FAME ^1^	PC3-EVs (*n* = 8) ^2^	LNCaP-EVs (*n* = 8)^2^
C14:0	4.38 ± 1.17	5.27 ± 0.25
C16:0	33.32 ± 0.74	35.62 ± 0.96
6*trans*-C16:1	0.10 ± 0.02	0.48 ± 0.03 ***
6*cis-C*16:1 n-10	6.10 ± 1.59	8.13 ± 0.28
9*cis*-C16:1 n-7	1.10 ± 0.26	1.59 ± 0.08
C18:0	12.35 ± 0.52	15.34 ± 0.66 **
9*trans*-C18:1	0.18 ± 0.04	0.22 ± 0.04
8*cis*-C18:1 n-10	2.85 ± 0.67	4.32 ± 0.27 *
9*cis*-C18:1 n-9	30.83 ± 4.41	15.43 ± 0.66 **
11*cis*-C18:1 n-7	1.21 ± 0.08	1.57 ± 0.24
5*cis*,8*cis*-C18:2 n-10	0.25 ± 0.03	0.62 ±0.07 ***
mono-trans C18:2 n-6	0.15 ± 0.03	0.20 ± 0.06
C18:2 n-6	3.43 ± 0.50	2.91 ± 0.18
C20:3 n-6	0.49 ± 0.08	0.98 ± 0.16 *
C20:4 n-6	0.70 ± 0.15	2.05 ± 0.13 ***
mono-trans C20:4	0.05 ± 0.02	0.08 ± 0.02
C20:5 n-3	0.29 ± 0.05	0.55 ± 0.09 *
C22:5 n-3	0.54 ± 0.14	1.41 ± 0.19 **
C22:6 n-3	1.68 ± 0.24	3.22 ± 0.23 ***
SFA	50.05 ± 1.50	56.23 ± 0.61 **
MUFA	42.08 ± 2.25	31.05 ± 0.49 ***
PUFA	7.39 ± 0.94	11.74 ± 0.41 ***
n-6	4.62 ± 0.57	5.94 ± 0.22 *
n-3	2.51 ± 0.38	5.18 ± 0.32 ***
n-6/n-3	1.97 ± 0.17	1.18 ± 0.08 ***
n-10	9.20 ± 2.23	13.07 ± 0.50
Total *trans*	0.48 ± 0.08	0.98 ± 0.09

^1^ Identified by standard references and quantified as described in the Materials and Methods Section. Values are obtained in µg/mL considering the GC peak areas recognized and calibrated with standard references (corresponding to >98% of the total peaks of the chromatogram). ^2^ EVs = extracellular vesicles. Values are expressed in percentages relative to the sum of all the quantities of the recognized peaks ± standard error of the mean (S.E.M) from the analyses of *n* = 8 cell samples of each type; statistical significance is estimated: * *p*-value ≤ 0.05; ** *p*-value ≤ 0.003; *** *p*-value ≤ 0.0006. Details of the statistical analysis are reported in the Materials and Methods Section.

## Supplementary Materials

The following are available online at https://www.mdpi.com/2072-6694/12/4/900/s1. Figure S1: Representative GC chromatogram of fatty acid methyl esters obtained from PC3 membrane phospholipids. In the boxes, the enlargement shows the areas containing C16 MUFA (green box) and 8*cis*-C18:1, 5*cis*, 8*cis*-C18:2 (purple box). Figure S2: Representative examples of FAME analyses coming from phospholipids of (A) (LNCaP cells), (B) (EVs LNCaP) and (C) (EVs PC3). Figure S3: Representative GC/MS analyses of FAME mixture obtained from membrane phospholipids of the (A) (PC3) and (B) (LNCaP) after DMSD derivatization following the protocol described in the Materials and Methods Section; GC/MS traces focus on the chromatographic region containing the FAME DMDS adducts of: 6*cis*-C16:1, 9*cis*-C16:1, *cis*8-C18:1, *cis*9-C18:1 and *cis*5, *cis*8-C18:2; in the bottom, the box contains details of the diagnostic fragmentations of the DMDS adducts, and the color codes indicate these fragments and their detection in the samples. Figure S4: Characterization of EVs released by PC3 and LNCaP cells. EVs were isolated from cell culture media of PC3 or LNCaP cells by differential centrifugation (see the Materials and Methods Section). (A) Recovered EVs quantified as µg proteins/106 cells (mean + SD, *n* = 8). (B) Scanning electron micrographs of EVs. See the Materials and Methods Section for experimental details. (C) Cell lysates and EV preparations were separated by SDS-PAGE, electrotransferred and probed with the indicated positive and negative EV markers. See the Materials and Methods Section for experimental details. Figure S5F. Original Western blot analyses for the five EV markers. A-CD9; B-ALIX; C-CD81; D-ACTIN; E-CALNEXIN. See Materials and Methods for experimental details. Table S1: Main lipid classes detected in PC3 and LNCaP cell membranes and their corresponding EVs expressed in µg/mL and reported as mean ± S.E.M. In the brackets, results are reported expressed as percentage of the sum of all identified lipids (*EVs versus corresponding cells). Table S2: Membrane phospholipid fatty acids of PC3 cells, PC3-EVs, LNCaP cells and LNCaP-EVs expressed in μg/mL. These data were used for the values in Table 1, and in Table 2 were expressed as % relative quantitative (% rel. quant.). Table S3: Primers used for qRT-PCR.

## Abbreviations

CHO	Cholesterol
DMDS	Dimethyl disulphide
DTT	Dithiothreitol
GADPH	Glyceraldehyde 3-phosphate dehydrogenase
ELOVL	Elongation enzyme
EV	Extracellular vesicle
FADS	Fatty acid desaturase
FAME	Fatty acid methyl esters
FBS	Fetal bovine serum
GC	Gas chromatography
HPLC	High-performance liquid chromatography
MUFA	Monounsaturated fatty acids
PBS	Phosphate buffered saline
PC	Phosphatidyl choline
PE	Phosphatidyl ethanolamine
PS	Phosphatidyl serine
PL	Phospholipid
PUFA	Polyunsaturated fatty acids
qRT-PCR	quantitative real Time polymerase chain reaction
SFA	Saturated fatty acids
SF	Sphingomyelin
SCD	Stearoyl CoA desaturase
TLC	Thin layer chromatography
