# Supplementary material for: The n-10 Fatty Acids Family in the Lipidome of Human Prostatic Adenocarcinoma Cell Membranes and Extracellular Vesicles
Source: Cancers (Basel). 2020 Apr 7;12(4):900. doi: 10.3390/cancers12040900 (PMC7226157; doi:10.3390/cancers12040900)
Supplement: Supplementary file 1 [file cancers-12-00900-s001.pdf]

cancers

MDPI

Article

The n-10 fatty acids family in the lipidome of human prostatic adenocarcinoma cell membranes and extracellular vesicles

Carla Ferreri, Chryssostomos Chatgilialoglu, Sandra Buratta, Lorena Urbanelli, Eva Costanzi, Carla Emiliani, and Anna Sansone

Table S1. Main lipid classes detected in PC3 and LNCaP cell membranes and their corresponding EVs expressed in µg/mL and reported as mean ± SEM. In the brackets are reported results expressed as percentage of the sum of all identified lipids (*EVs *vs* corresponding cells, p<0.05).

Lipids§	PC3 cells (*n* = 8) µg/mL	PC3-EVs (*n*=8) µg/mL	LNCaP cells (*n*=8) µg/mL	LNCaP-EVs (*n*=8) µg/mL
CHO	30.37±8.35 *(6.5±0.7%)*	18.63±3.26 *(12.7±3.2%*)*	32.5±3.5 *(5.8±0.4%)*	22.41±6.29 *(15.5+1.5%*)*
SM	48.58±8.35 *(10.3±1.6%)*	29.94±17.52 *(10.1±12.2%)*	62.40±10.73 *(11.3±2.0%)*	19.8±8.69 *(11.6±2.8%)*
PE	161.11±32.11 *(34.8±3.3%)*	23.92±7.23 *(16.6±4.3%*)*	203.36±36.00 *(36.5±4.2%)*	51±34.55 *(26.5±10.0%*)*
PS	23.42±4.18 *(5.2±1.3%)*	38.65±11.03 *(26.1±5.6%*)*	28.79±3.29 *(5.2±0.5%)*	29.22±5.35 *(18.2±5.1%*)*
PC	204.27±36.56 *(43.2±5.5%)*	37.05±14.21 *(25.5±8.6%*)*	230.61±8.14 *(41,1±2.7%)*	48.5±10.35 *(30.2±7.3%*)*

§ Abbreviations: CHO = cholesterol; SM = sphingomyelins; PE = phosphatidyl ethanolamine; PS = phosphatidyl serine; PC = phosphatidyl choline. Lipid classes are identified by the standard references as described in Materials and Methods.

Table S2. Membrane phospholipid fatty acids of PC3 cells, PC3-EVs, LNCaP cells and LNCaP-EVs expressed in µg/mL. These data were used for the values in Tables 1 and 2 expressed as % relative quantitative (% rel. quant.).

FAME [1]	PC3 cells (*n* = 8)	PC3-EVs (*n* = 8)	LNCaP cells (*n* = 8)	LNCaP-EVs (*n* = 8)
C14:0	12.15±1.26	5.04±1.30	11.36±0.68	6.97±0.38
C16:0	123.18±4.53	38.82±1.46	110.76±3.53	47.01±1.40
6*trans*-C16:1	1.35±0.15	0.11±0.03	1.83±0.08	0.63±0.04
6*cis*-C16:1 n-10	27.53±1.33	7.02±1.78	25.65±1.01	10.74±0.43
9 *cis*-C16:1 n-7	8.75±0.65	1.31±0.35	6.44±0.19	2.10±0.11
C18:0	40.51±1.87	14.48±1.00	37.77±0.86	20.21±0.83
9*trans*-C18:1	0.35±0.04	0.21±0.05	0.36±0.07	0.30±0.05
8*cis*-C18:1 n-10	22.31±2.87	3.32±0.77	14.97±0.39	5.73±0.41
9*cis*-C18:1 n-9	72.26±2.47	36.00±5.27	61.30±1.30	20.36±0.91
11*cis*-C18:1 n-7	14.61±1.15	1.42±0.13	12.80±0.17	2.05±0.29
5*cis*,8*cis*-C18:2 n-10	1.74±0.11	0.30±0.05	1.71±0.14	0.82±0.09
mono-trans C18:2 n-6	1.02±0.18	0.17±0.03	0.80±0.08	0.27±0.08
C18:2 n-6	9.21±0.68	3.98±0.61	6.49±0.33	3.86±0.27
C20:3 n-6	6.02±0.62	0.57±0.09	5.67±0.58	1.30±0.20
C20:4 n-6	10.66±0.89	0.85±0.20	13.15±0.41	2.71±0.18
mono-trans C20:4	0.36±0.03	0.05±0.02	0.37±0.15	0.10±0.03

C20:5 n-3	1.75±0.21	0.33±0.05	1.24±0.23	0.71±0.12
C22:5 n-3	6.58±0.70	0.63±0.16	5.06±0.52	1.87±0.27
C22:6 n-3	11.07±0.70	1.97±0.31	11.51±0.71	4.26±0.34
SFA	175.84±4.53	58.34±2.52	159.89±4.35	74.19±1.19
MUFA	145.45±4.63	49.06±3.02	121.16±2.20	40.97±0.86
PUFA	47.02±1.57	8.63±1.20	44.83±1.62	15.53±0.69
n-6	25.89±1.36	5.40±0.73	25.31±1.01	7.86±0.34
n-3	19.39±0.56	2.93±0.46	17.81±1.36	6.85±0.47
n-6/ n-3	1.34±0.07	1.97±0.17	1.52±0.19	1.18±0.08
n-10	51.58±3.82	10.64±2.52	42.34±1.22	17.29±0.81
Total *trans*	3.08±0.27	0.56±0.10	3.35±0.17	1.30±0.12

[1] identified by standard references and quantified as described in Materials and Methods. Values are obtained in μg/mL considering the GC peak areas recognized and calibrated with standard references (corresponding to >98% of the total peaks of the chromatogram). Values are expressed in μg/mL ± Standard Error of the Mean (s.e.m) from the analyses of n=8 cell samples of each type.

Table S3. Primers used for qRT-PCR.

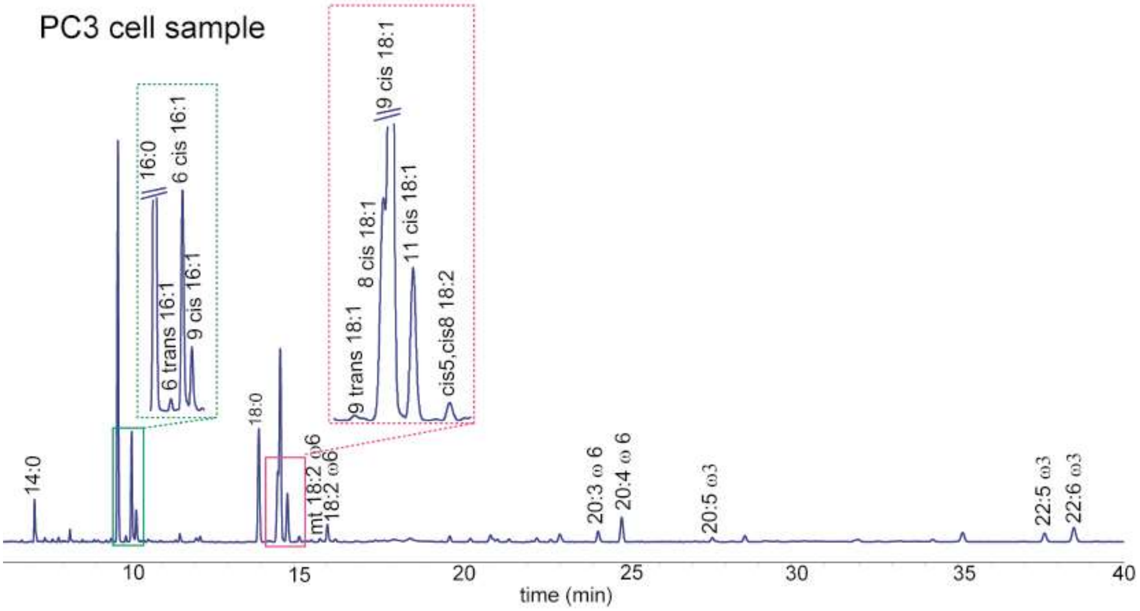

Figure S1. Representative GC chromatogram of fatty acid methyl esters obtained from PC3 membrane phospholipids. In the boxes the enlargement of the areas containing C16 MUFA (green box) and 8*cis*-C18:1, 5*cis*,8*cis*-C18:2 (purple box).

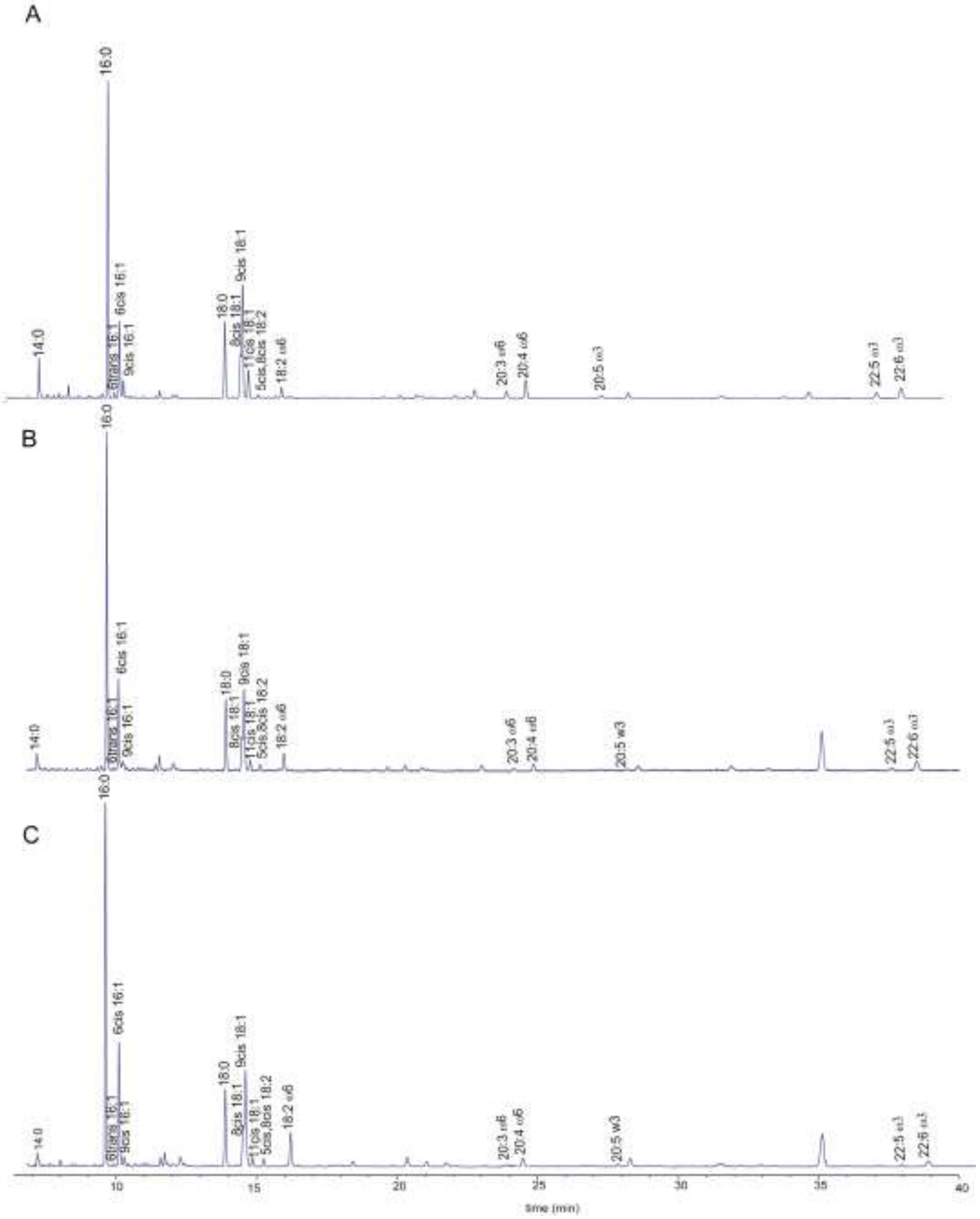

Figure S2. Representative examples of FAME analyses coming from phospholipids of A, (LNCaP cells), B (LNCaP-EVs) and C (PC3-EVs).

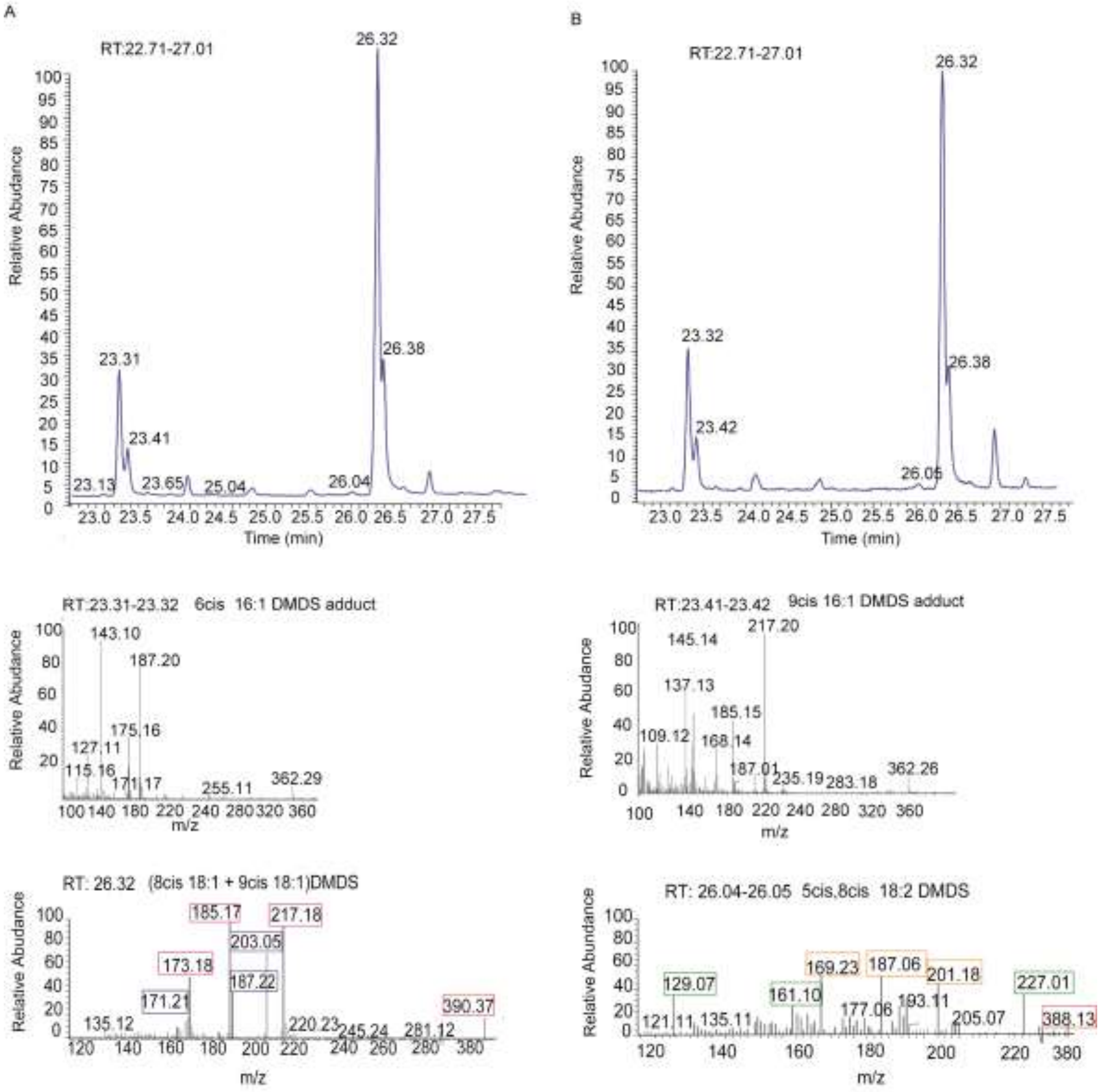

DMDS adducts	ω fragment	Δ fragment	Δ fragment-32
6cis 16:1	187.20	175.16	143.10
9cis 16:1	145.14	217.20	185.15
8cis 18:1	187.22	203.05	171.21
9cis 18:1	173.18	217.18	185.17
5cis,8cis 18:2	227.01-187.06	161.10-201.18	129.07-169.23

Figure S3. Representative GC/MS analyses of FAME mixture obtained from membrane phospholipids of the A, (PC3) and B (LNCaP) after DMSD derivatization following the protocol described in Materials and Methods; GC/MS traces focus on the chromatographic region containing the FAME DMDS adducts of: 6*cis*-C16:1, 9*cis*-C16:1 , *cis*8-C18:1, *cis*9-C18:1 and *cis*5,*cis*8-C18:2; in the bottom, the box contains details of the diagnostic fragmentations of the DMDS adducts, and the color codes indicate these fragments and their detection in the samples.

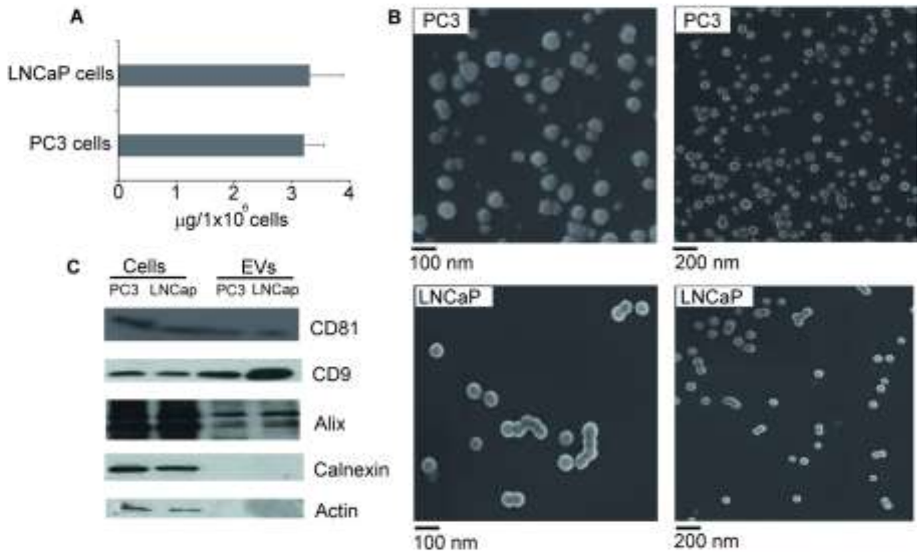

Figure S4. Characterization of EVs released by PC3 and LNCaP cells. EVs were isolated from cell culture. Media of PC3 or LNCaP cells by differential centrifugation (see Materials and Methods). A) Recovered EVs quantified as μg proteins/10^6 cells (mean+S.D., n=8). B) Scanning electron micrographs of EVs. See Materials and Methods for experimental details. C) Cell lysates and EV preparations were separated by SDS-PAGE, electrotransferred and probed with the indicated positive and negative EV markers. See Materials and Methods for experimental details.

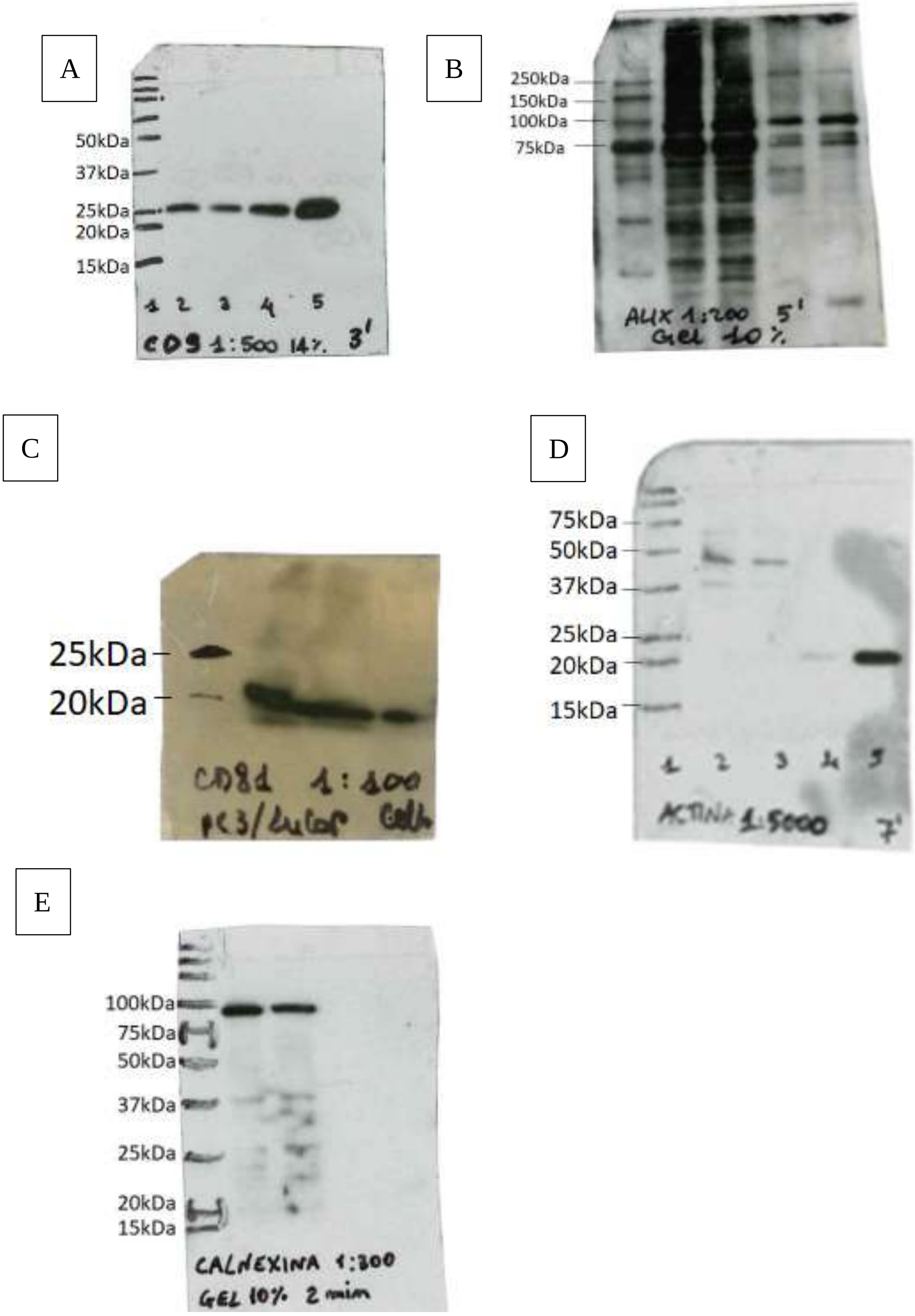

Figure S5. Original Western blot analyses for the five EV markers. A- CD9; B- ALIX; C- CD81; D- ACTIN; E- CALNEXIN. See Materials and Methods for experimental details.
